# Supplementary figures and images for: Reduced-port laparoscopic distal gastrectomy in obese gastric cancer patients
Source: PLoS One. 2021 Aug 5;16(8):e0255855. doi: 10.1371/journal.pone.0255855 (PMC8341504; doi:10.1371/journal.pone.0255855)

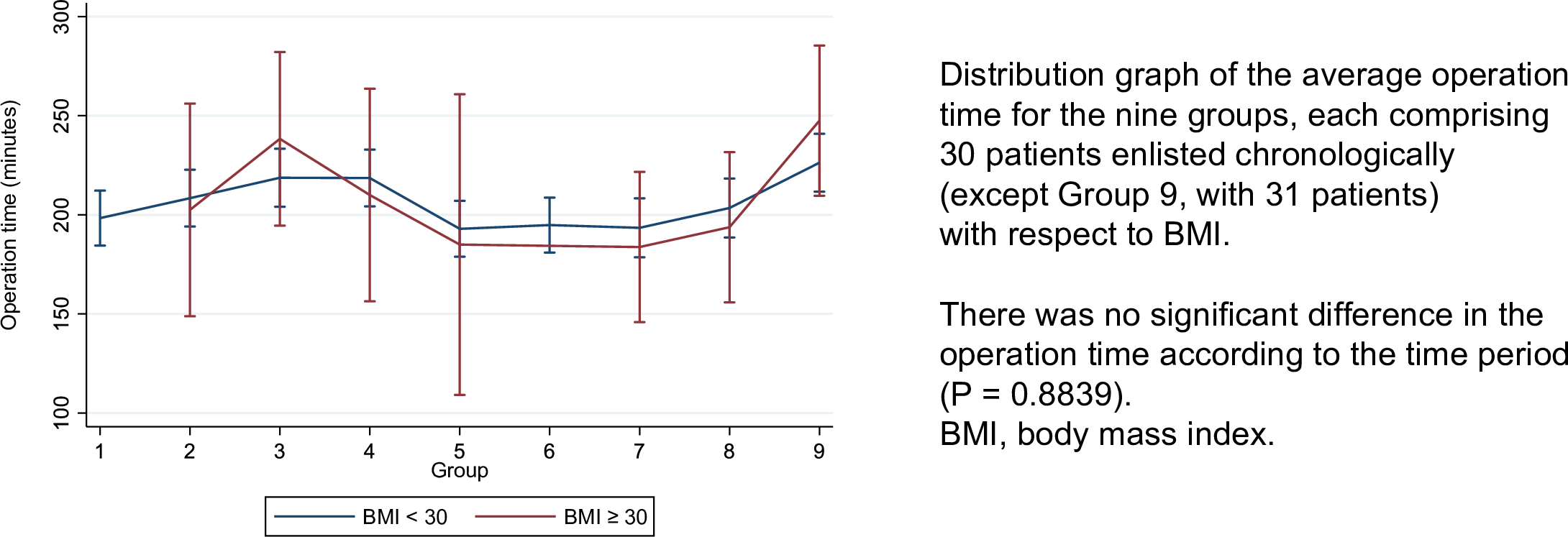

Supplement: S1 Fig — (TIF) [file pone.0255855.s001.tif]
