# Supplementary material for: Reduced-port laparoscopic distal gastrectomy in obese gastric cancer patients
Source: PLoS One. 2021 Aug 5;16(8):e0255855. doi: 10.1371/journal.pone.0255855 (PMC8341504; doi:10.1371/journal.pone.0255855)
Supplement: Attachment — Submitted filename: Response to Reviewers.docx [file pone.0255855.s002.docx]

PONE-D-21-19851
Reduced-port laparoscopic distal gastrectomy in obese gastric cancer patients
PLOS ONE

*Dear reviewers,*

*Thank you very much for the two expert reviews. With the help of your advice, we are happy that our insufficient study can have a higher level of completeness. We will work harder until it is published on PLOS ONE. Then we respond to your comments.*

Reviewer #1: In this research article, the authors aimed to evaluate the short-term surgical outcomes and investigate the feasibility and safety of reduced-port laparoscopic distal gastrectomy in obese patients with gastric carcinoma. They conducted a retrospective study of 271 gastric cancer patients who underwent reduced-port laparoscopic distal gastrectomy, where they divided the patients into two groups as non - obese and obese in order to compare and analyse their surgical outcomes. They concluded that reduced-port laparoscopic distal gastrectomy could be performed safely in obese gastric cancer patients. The study is well designed and adequately written. I congratulate the authors for their successful work.

***Answer*** *: Thank you for your favorable reception of our research. I hope to have another opportunity to meet you with better study in the future.*

Reviewer #2: Comments to the manuscript:

I think that it is an interesting paper to reflect the preliminary results of reduced-port laparoscopic gastrectomy in patients with gastric malignancy (with distal gastrectomy), comparing obese patients with non-obese patients. However, as the authors reflect in the limitations of the study, it is a retrospective study with few patients, so I would accept it in a short and more summarized format.

I further state some considerations that I believe should be better clarified in this manuscript:

1. In the Methodology section, what were the exclusion criteria for the study? The authors state that this surgery was offered to all patients a priori, but they do not state whether patients were initially excluded for reasons other than obesity. It should also be specified at what tumor stage this surgery was offered to patients with gastric neoplasia. It is only mentioned that surgery was considered when it was technically feasible, but it should be better detailed in cases where this surgical approach was not considered.

***Answer*** *: We apologize that the exclusion criteria was vaguely presented. We corrected the text as suggested. There were no specific criteria other than clinical stage and total gastrectomy. Among the patients who undergone total gastrectomy, obese patient was very rare, and we thought that ‘total gastrectomy’ and ‘distal gastrectomy’ should be analyzed separately, so we limited this study to ‘distal gastrectomy’. We appreciate your comments.*

2. On the other hand, other aspects that seem to be better in this surgical approach with respect to the conventional laparoscopic approach could have been evaluated, such as cosmetic satisfaction and early oral intake. In addition, tumor recurrence and mean follow-up time are variables that I consider important to analyze.

***Answer*** *: Thank you for recognizing the advantages of our surgical approach. As you suggest, we plan to follow up long-term surgical outcomes and analyze them. Since this takes time, unfortunately, this our study only reflects the short-term results.*

3. Given the previous studies, which reflect a longer surgical time in obese patients with conventional laparoscopic approach, it would be interesting to compare, as a future project, obese patients with conventional laparoscopic approach versus reduced-port laparoscopic gastrectomy.

***Answer*** *: Thank you for suggesting a good direction for the next project. This will be planned as multi-center study, and if there is an opportunity, I hope you to review our new study.*

*Thank you for your consideration. I look forward to hearing from you.*

*Sincerely,*

*Dong Yi Kim*
